# Editorial: Population Genetics of Worldwide Jewish People

**DOI:** 10.3389/fgene.2017.00101

**Published:** 2017-07-28

**Authors:** Eran Elhaik

**Affiliations:** Department of Animal and Plant Sciences, University of Sheffield Sheffield, United Kingdom

**Keywords:** Jewishness, ancestry, genomes, Ashkenazic Jews, Sephardic Jews

## Background

Stephen Jay Gould remarked that “the most erroneous stories are those we think we know best—and therefore never scrutinize or question” (Gould, 1996). In the past, shamans and priests were believed to have omnipotence in controlling nature, man, and fate. As guardians of history and memory, they developed captivating narratives that bounded nature, religion, and mythology and aspired humans to continue their efforts to tame the natural and supernatural worlds. Nowadays, scientists have adopted the traditional role of the shamans and, grievously, some of their inclination to narratives (Sand, 2015).

In reconstructing the past from the distribution of genetic variation, population geneticists oftentimes rely on narratives. To decide between scenarios, geneticists have a multitude of accessories ranging from evolutionary theories to advanced computational tools applicable to modern and ancient genomes (Veeramah and Hammer, 2014; Morozova et al., 2016). In their efforts to understand human origins, geneticists also reach out to other disciplines like anthropology, linguistics, archeology, and history. However, as with any historical reconstruction, the inferred past remains a subject of controversy due to the subjectivity of the data, tools, assumptions, and, most importantly, the narratives that guided the scientist (Sand, 2015). Genetic studies of Jewish communities are especially vulnerable to such controversies as these communities have adopted various narratives since their inception (e.g., Patai and Patai, 1975; Kirsh, 2003, 2007; Kahn, 2005; Falk, 2006; Sand, 2009).

A narrative may meet its demise in a number of ways. It can evolve into a new narrative, usually by assimilating elements of other narratives, it can evolve by “drift” and eventually be replaced by a fitter variant, or it can be surrendered to scientific scrutiny that may either prove or dismiss it as fictitious.

This is now the case with two central Judeo-Christian narratives: the first, proposed less than two centuries ago by historian Heinrich Graetz, depicts the origin of modern-day Jews as the lineal descendants of the Biblical Judaeans. This narrative lacks historical (Sand, 2009) and linguistic (Wexler, 1993, 2011) evidence. The second, rooted in first century Christian myths that were internalized by Jewish scholars, alludes to the “Roman Exile” that followed the destruction of Herod's temple (70 A.D.) and introduced a massive Jewish population to Roman lands (Yuval, 2006). Such a population transplant, however, also lacks historical and linguistic support (Horon, 2000; Yuval, 2006; Sand, 2009; Wexler, 2016).

**Figure 1:**
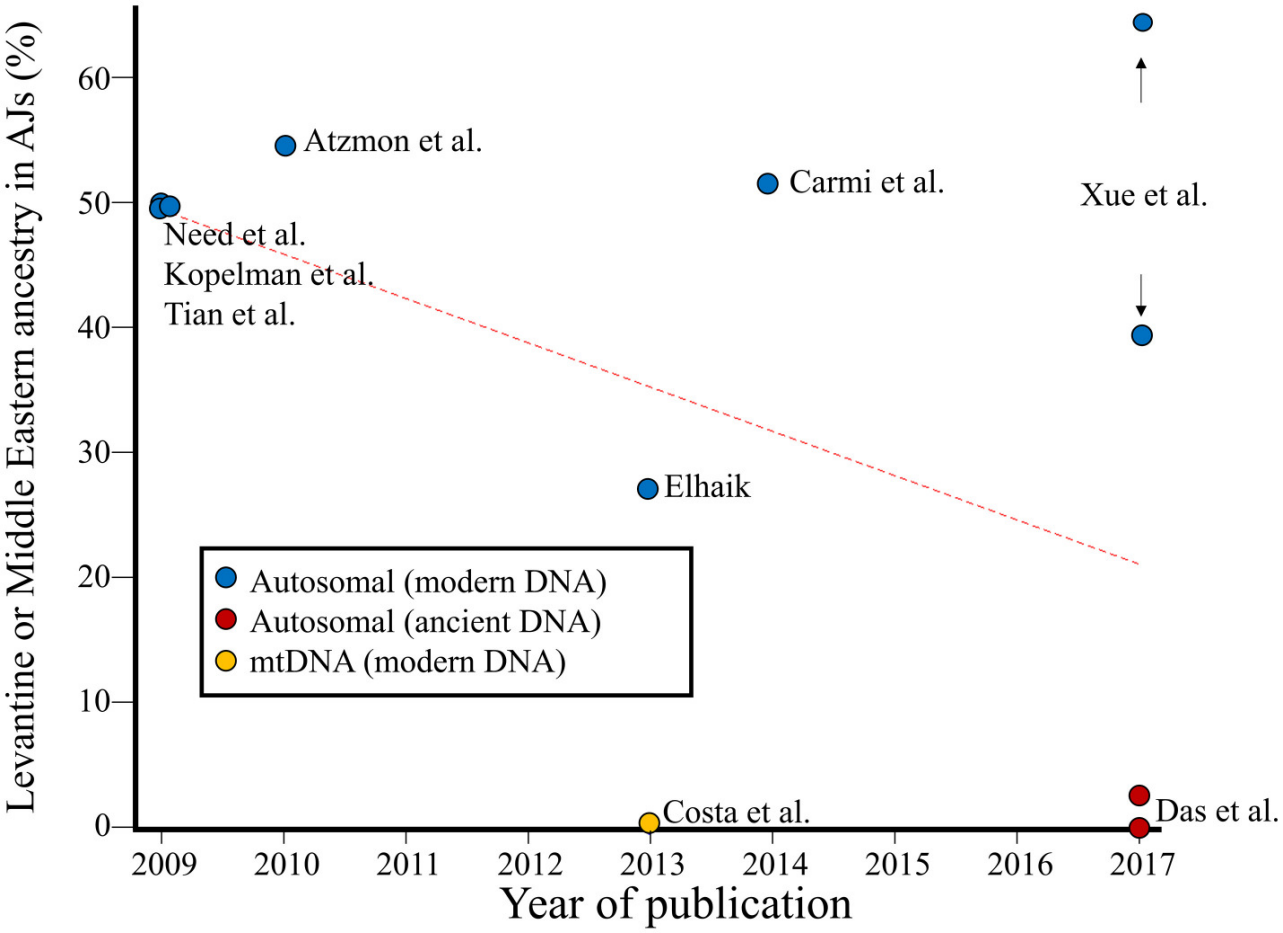
The Levantine or Middle Eastern ancestry of Ashkenazic Jews over time. Nine genomic estimates of the Levantine ancestry in AJs (2009–2017) (Kopelman et al., 2009; Need et al., 2009; Tian et al., 2009; Atzmon et al., 2010; Costa et al., 2013; Elhaik, 2013; Carmi et al., 2014; Das et al., 2016; Xue et al., 2017) derived from autosomal, whole genome, or mtDNA data are shown. Studies reporting “intermediate” percentage between Middle Eastern and European populations are shown as 50% Middle Eastern ancestry. The percentage of Levantine ancestry among AJs shows a decreasing trend over time (*r* = −0.49) with the most recent estimates being close to zero.

Most of the genetic studies on Jews focused on Ashkenazic Jews (AJs). The first genetic study that challenged the Levantine origin of AJs argued that such an origin has only been upheld and “replicated” due to the false dichotomy fallacy and that a Caucasus origin, never truly explored, explains the data better (Elhaik, 2013). A follow-up study (Costa et al., 2013) reported that at least 90% of Ashkenazic maternal ancestry is indigenous to Europe and likely originated through conversion of local populations with the remaining ancestries having East Asian or unidentified origins. These finding are supported by ancient DNA evidence showing 0–3% Levantine ancestry and a dominant Iranian ancestry (88%) in modern-day AJs (Das et al.). Interestingly, this evidence explains the higher estimates of Middle Eastern ancestry ranging from 27 to 65% (Figure 1) in that previous analyses either considered Iran and the Caucasus as part of the “Middle East,” thereby inflating the proportion of Middle Eastern ancestry, or compared AJs to Palestinians, themselves a population with 40% non-Levantine ancestry that increased their similarity to AJs (Das et al.). The second narrative has recently been revived due to the genetic similarity between AJs and south European populations (Xue et al., 2017). However, this similarity can be explained by the Greco-Roman origin of AJs who lived along the shores of the Black Sea in “ancient Ashkenaz” during the early centuries A.D. (Das et al., 2016), which is supported by historical (Harkavy, 1867) and linguistic evidence (Das et al., 2016). In light of these findings (Figure 1), Ostrer's proposal that land disputes in the Middle East should be decided by the proportion of Middle Eastern ancestry in one's genome (Ostrer, 2012) is regrettable and underlies the danger in developing policy based on ill-founded narratives.

These are not the only Jewish narratives in question. Over the past years, historical, theological, linguistic, and genetic narratives have all been challenged and replaced by new theories (Patai, 1990; Wexler, 1993, 1996; Finkelstein and Silberman, 2002; Sand, 2009; Finkelstein, 2013; Kohler, 2014; Das et al., 2016; Elhaik). This was to be expected since, dismantling these narratives not only undermined their historical basis but also rendered any insights about the Judaeans gained by studying modern-day Jews erroneous.

To reflect upon the exhilarating progress in the youngest of these fields—population genetics—this *Frontiers*' topic aimed to bring the most updated findings and perspectives. The first paper of this topic (Tofanelli et al.) examines the “Cohen gene” hypothesis originated by Skorecki et al. (1997). In that study, the authors reported that individuals with the surname Cohen spotted in Canada, the UK, and Tel Aviv's beaches (Goldstein, 2008) exhibit genetic differences from the general Israeli population in their Y chromosome. Skorecki and colleagues claimed that these differences evidenced their descent from ancient Judaean high priests, although ancient priests were never sampled. Tofanelli et al. showed that the “Cohen gene” narrative lacks biological support and criticized the use of haplotype motifs as reliable predictor of “Jewishness.” Nogueiro et al. studied the origin of Portuguese Sephardic Jews. The authors reported that the genetic diversity of uniparental markers alludes to the complexity of the demographic processes underlying the genetic pool of the Portuguese Crypto-Jews' descendants, which likely involve introgression from and admixture with Iberian populations. These findings were called into question for being interpreted within an *a priori* narrative depicting Portuguese Crypto-Jews as a reproductive isolate (Marcus et al.). Falk's perspective pulled the rug from under the field of Jewish genetics, arguing that thus far no Jewish markers were found, which highlights the imminent question—who are the people being studied and what is their relatedness to the ancient Judaeans, if any? Elhaik developed Falk's postulate into a blind-test and invited members of the public, academia, and industry who claimed they can genomically distinguish Jews from non-Jews to prove their claims. Failing to satisfy the terms of the test and explaining why “Jewish biomarkers” are unlikely to exist, Elhaik concluded that all the findings concerning Jewish genetics should be critically evaluated.

The conclusions of these studies are innovative. The abandonment of the Levantine origin of Jews prompts new questions concerning the origin of various Jewish communities, the gene flow experienced with other communities, and the fate of the ancient Judaeans, which some authors discuss. The work presented here leaves aside many other narratives that should also be reevaluated, such as the purported absence of alcoholics among Jews (Keller, 1970), thought to have a genetic basis (Bray et al., 2010), whereas in reality alcoholism in Israel is a major concern (Efrati, 2014). We hope that articles published under this topic would be valuable for future scholarship.

## Author contributions

The author confirms being the sole contributor of this work and approved it for publication.

### Conflict of interest statement

EE is a consultant for DNA Diagnostic Centre.
